# Supplementary material for: RSPO3 impacts body fat distribution and regulates adipose cell biology in vitro
Source: Nat Commun. 2020 Jun 3;11:2797. doi: 10.1038/s41467-020-16592-z (PMC7271210; doi:10.1038/s41467-020-16592-z)
Supplement: Supplementary file 1 — Supplementary Information [file 41467_2020_16592_MOESM1_ESM.pdf]

RSPO3 impacts body fat distribution and regulates adipose cell biology in vitro

Loh et al.

Supplementary Information

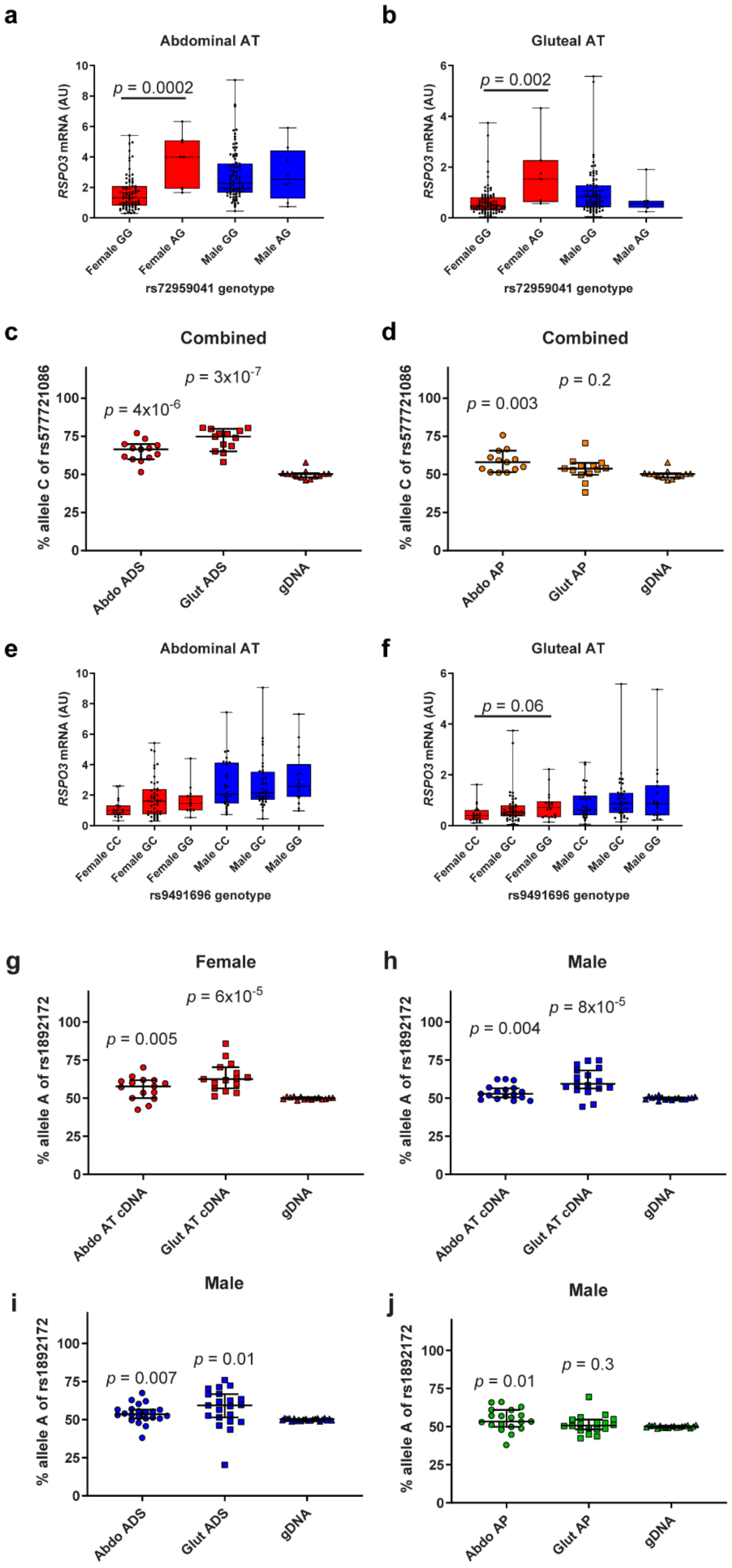

Supplementary Fig. 1. Effects of WHRadjBMI-increasing alleles at *RSPO3* on *RSPO3* expression in whole and fractionated AT. (**a-b**) Normalised RSPO3 mRNA levels in (**a**) SC

abdominal (Abdo) and (**b**) gluteal (Glut) fat biopsies from 102 women and 97 men, grouped by their rs72959041 genotype (WHRadjBMI increasing allele = A). (**c-d**) Allelic expression analysis of RSPO3 transcripts in abdominal and gluteal (**c**) isolated adipocyte (ADS) and (**d**) cultured AP cDNAs from 13 heterozygous carriers at rs72959041 (10 females and 3 males). The proportion of RSPO3 cDNA and gDNA containing the (WHR-increasing) allele C of rs577721086 (r^2 = 0.98 with rs72959041-A) is quantified on the y-axis. (**e-f**) Normalised RSPO3 mRNA levels in (**e**) SC abdominal (Abdo) and (**f**) gluteal (Glut) fat biopsies from 94 women and 89 men, grouped by their rs9491696 (WHRadjBMI increasing allele = G) genotype. All individuals analysed are homozygous for the rs72959041-G (non-risk) allele. (**g-j**) Allelic expression imbalance of RSPO3 was assessed by qRT-PCR in (**g-h**) abdominal and gluteal AT cDNAs from 15 females and 17 males, and in (**i-j**) abdominal and gluteal (**i**) isolated adipocyte (ADS) (*n* = 22 pairs), and (**j**) cultured AP (*n* = 19 pairs) cDNAs, from males, heterozygous at rs9491696 and homozygous for the rs72959041-G allele. The proportion of total cDNA and gDNA containing the WHRadjBMI-increasing allele A of rs1892172 (r^2 = 0.84 with rs9491696-G) is quantified on the y-axis. (**a**, **b**, **e**, **f**) Univariate analyses were adjusted for age and BMI, and corrected for multiple testing (Sidak) (**e**, **f**). Box and whisker plot: center line, median; box limits, upper and lower quartiles; and whiskers, maximum and minimum values. (**c**, **d**, **g-j**) Error bars are median values with 95% confidence intervals. Statistical significance was assessed by two-tailed paired Student's t-tests vs. gDNA. Source data are provided as a Source Data file.

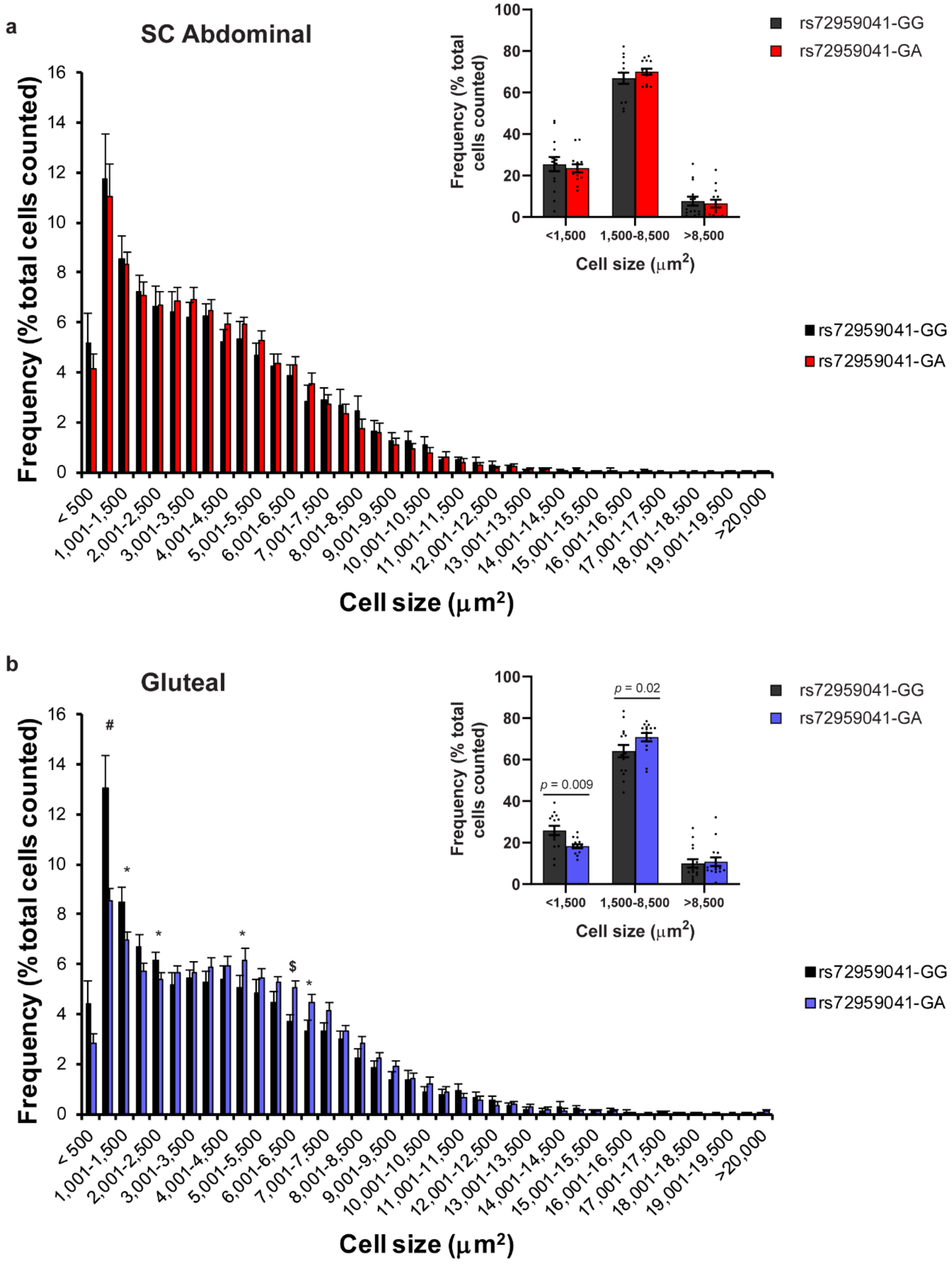

Supplementary Fig. 2. Adipocyte cell size distribution in females grouped by rs72959041 genotype. Cell size frequency of adipocytes from (**a**) SC abdominal and (**b**) gluteal AT histological sections from 14 and 15 pairs of age- and BMI-matched females, respectively,

grouped by rs72959041 genotype. Results are shown as relative frequency of adipocytes of different sizes (cross sectional area, μm^2). Inset graph is the simplified version of the main graph. More than 250 adipocytes were measured for each biopsy. Histogram data are expressed as means ± s.e.m.. Statistical significance was assessed by two-tailed paired Student's t-tests. $^*p \leq 0.05$, $^\#p < 0.01$, $^\$p < 0.001$. Source data are provided as a Source Data file.

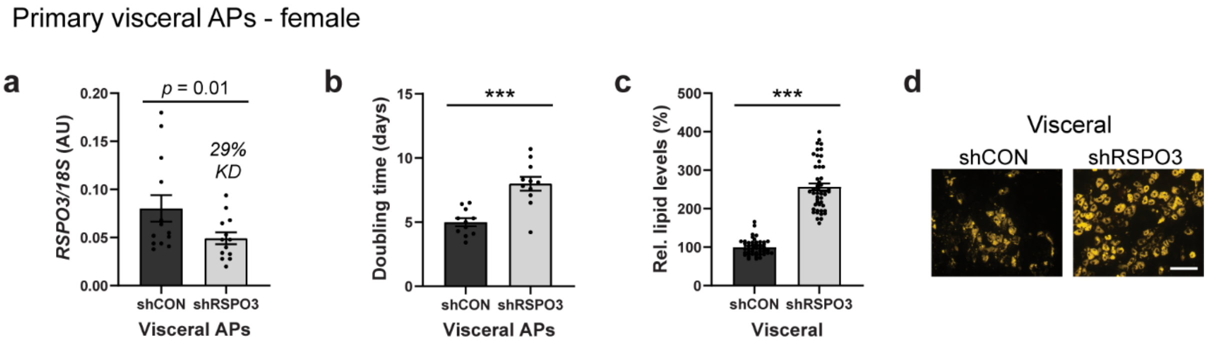

Supplementary Fig. 3. Effects of *RSPO3*-KD on proliferation and differentiation in primary visceral APs. (**a**) *RSPO3*-KD was confirmed by qRT-PCR (n = 13 experiments). Effects of *RSPO3*-KD on visceral AP (**b**) proliferation (n = 11 experiments) and (**c**) differentiation (two independent experiments, n = 24 replicates each). (**d**) Representative micrographs of AdipoRed-stained cells. Intracellular lipids are stained yellow. Scale bar = 200 µm. Histogram data are means ± s.e.m.. ***$p < 0.001$. qRT-PCR data were normalised to *18S*. Statistical significance was assessed by two-tailed paired (**a**, **b**) and unpaired (**c**) Student's t-tests. Source data are provided as a Source Data file.

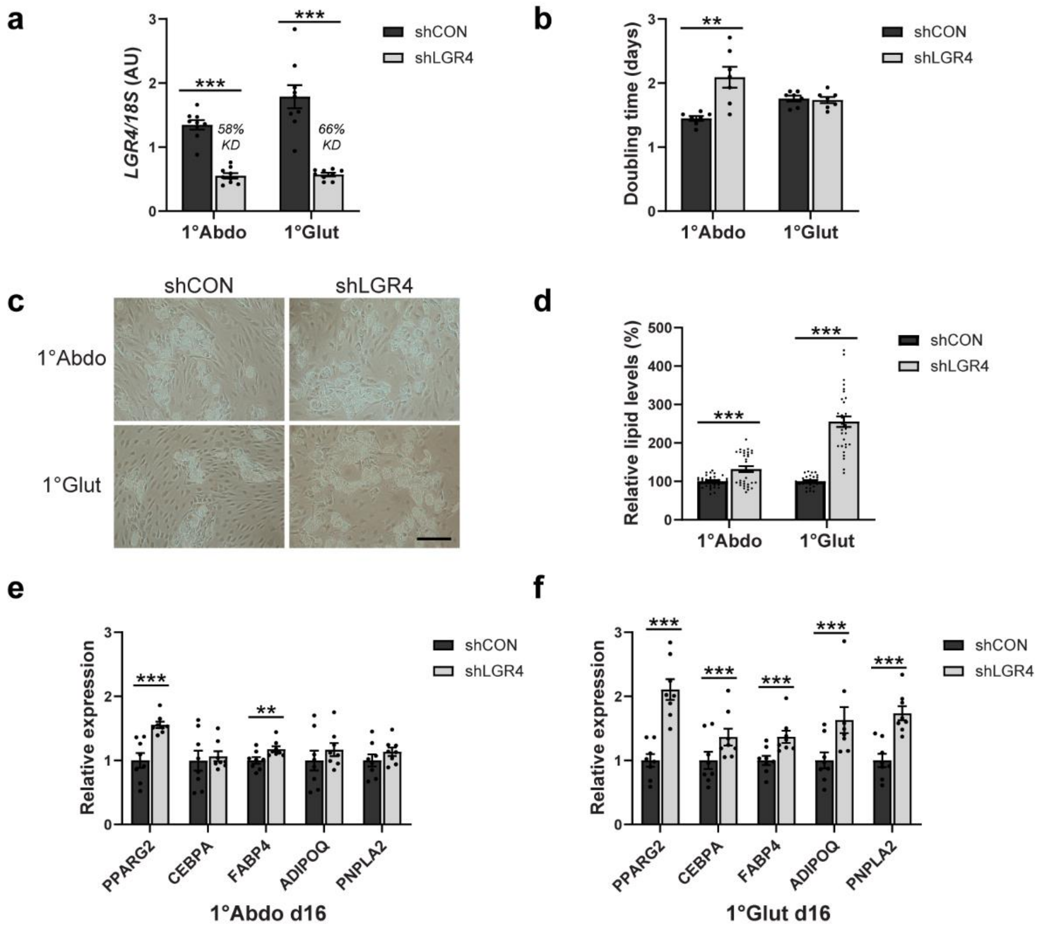

Supplementary Fig. 4. Effects of *LGR4*-KD in SC abdominal and gluteal APs on proliferation and adipogenesis. (**a**) *LGR4*-KD in primary (1°) APs was confirmed by qRT-PCR (n = 9 experiments). shCON = control, shLGR4 = *LGR4*-KD cells. (**b**) Doubling time of shCON and shLGR4 1°Abdo and 1°Glut APs (n = 7 experiments). (**c-f**) *LGR4*-KD leads to increased adipogenesis. (**c**) Representative micrographs of shCON and shLGR4 1°Abdo and 1°Glut cells following 14 days of adipogenic differentiation. Scale bar = 200 μm. Efficiency of adipogenesis was assessed with (**d**) AdipoRed staining (4 independent experiments, n = 8 replicates each, expressed as relative lipid levels) and (**e**) qRT-PCR of adipogenic genes (n = 8 experiments). Histogram data are means ± s.e.m.. $^{**}p < 0.01$, $^{***}p < 0.001$, shCON vs. shLGR4. Statistical significance was assessed by two-tailed paired (**a**, **b**, **e**, **f**) and unpaired (**d**) Student's t-tests. Source data are provided as a Source Data file.

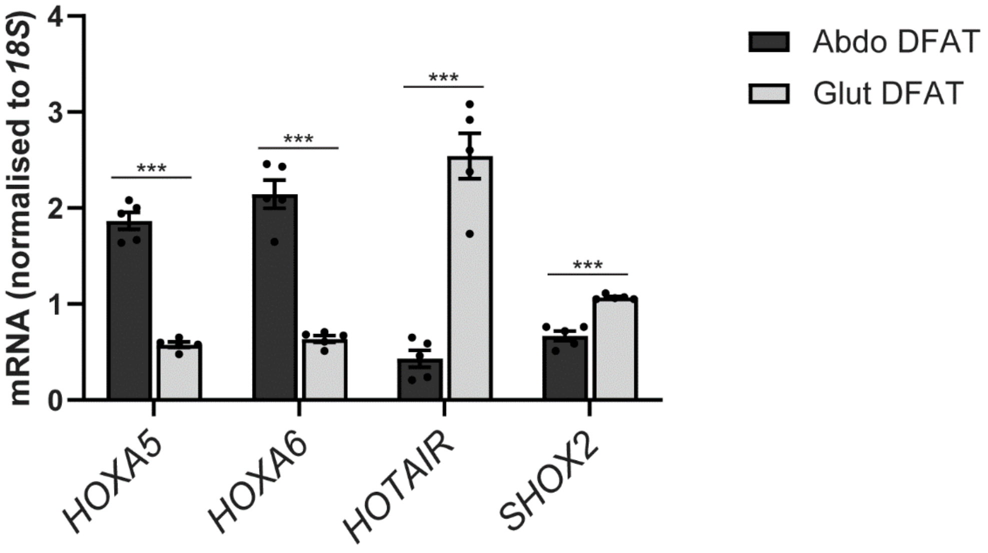

Supplementary Fig. 5. Abdominal and gluteal DFAT cells retain their depot-specific gene expression signatures. In vitro differentiated abdominal and gluteal DFAT cells (n = 5 independent experiments) were assessed by qRT-PCR for *HOXA5* and *HOXA6* which are more highly expressed in abdominal APs and mature adipocytes, and *HOTAIR* and *SHOX2*, which are more highly expressed in gluteal APs and adipocytes. Histogram data are means ± s.e.m.. ***$p < 10^{-7}$. qRT-PCR data were normalised to *18S*. Statistical significance was assessed by two-tailed paired Student’s t-tests. Source data are provided as a Source Data file.

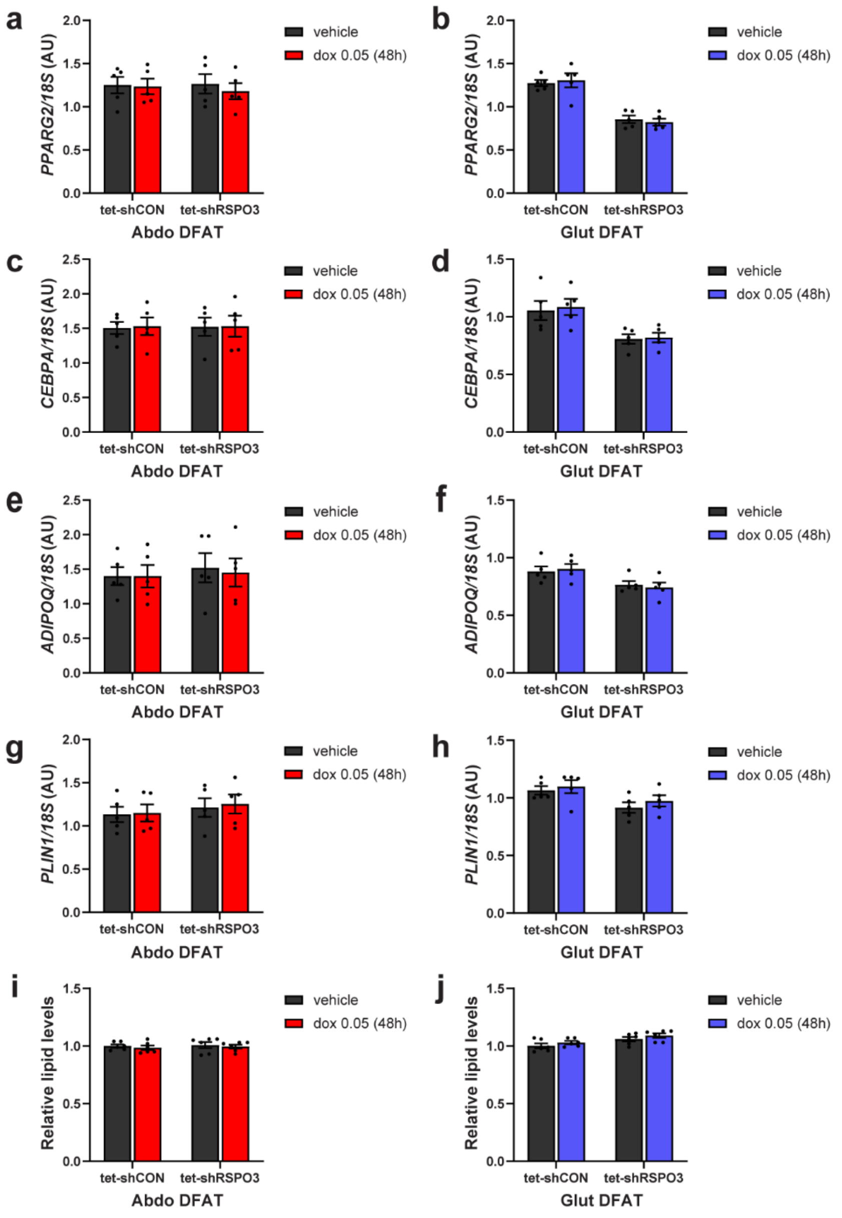

Supplementary Fig. 6. Effects of induced *RSPO3*-KD on adipogenic gene expression and lipid accumulation in in vitro differentiated DFAT adipocytes. qRT-PCR analysis of (**a-b**) *PPARG2*, (**c-d**) *CEBPA*, (**e-f**) *ADIPOQ* and (**g-h**) *PLIN1*, in cDNAs from DFAT cells at day 15 of adipogenic differentiation following ~48 hours treatment with 0.05 μg ml^{-1} doxycycline vs. vehicle in hormone-free basal media (n = 5 experiments). (**i-j**) Adipogenesis, assessed by AdipoRed staining, was not different between groups (n = 6 replicates). Histogram data are means ± s.e.m.. qRT-PCR data were normalised to *18S*. Source data are provided as a Source Data file.

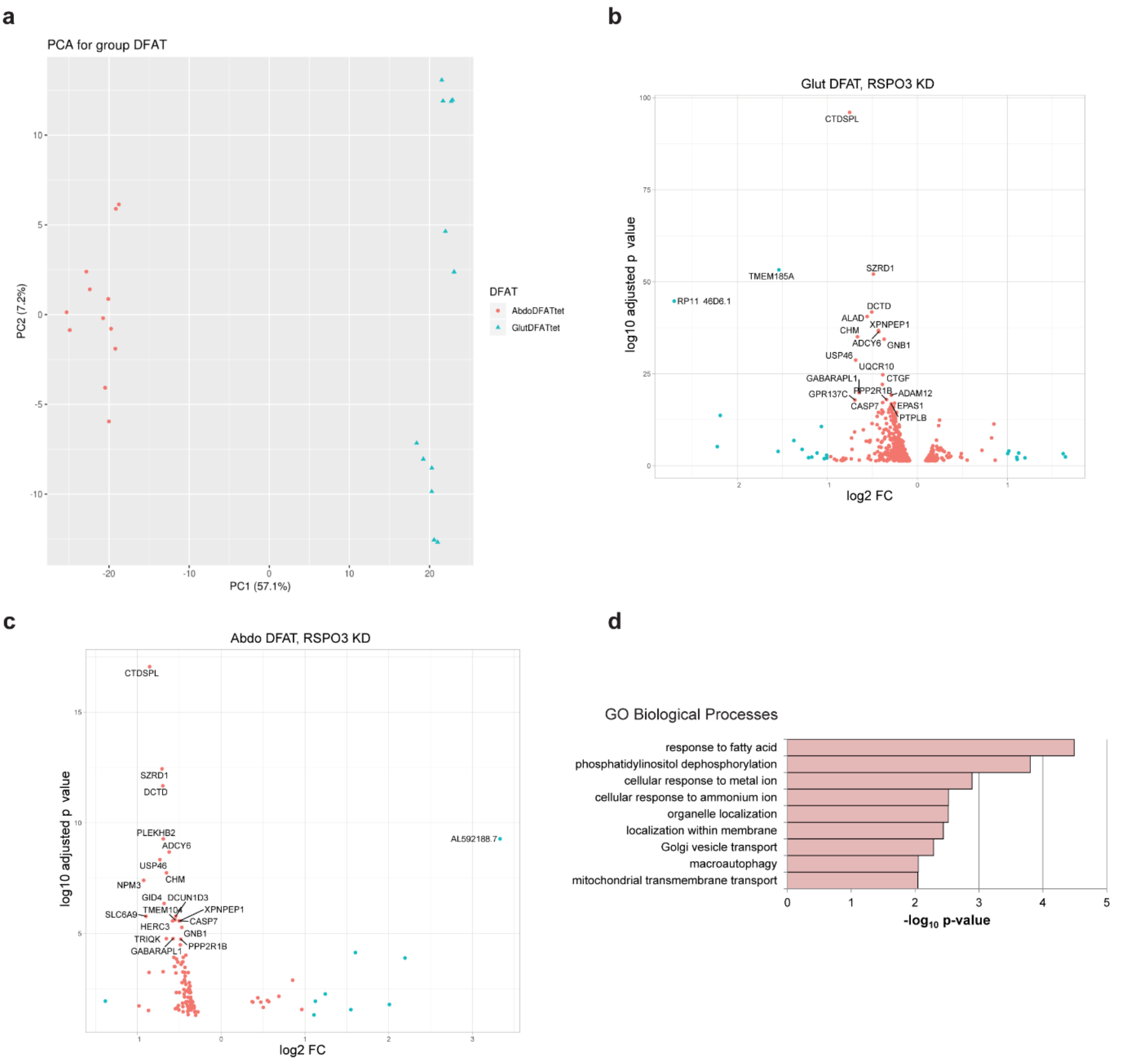

Supplementary Fig. 7. RNA-seq analysis of in vitro differentiated abdominal and gluteal DFAT cells following 48-hour doxycycline-induced *RSPO3*-KD. (**a**) Principal component analysis of RNA-seq data grouped by cell-type. (**b-c**) Volcano plots of the differentially expressed genes (DEGs) with *RSPO3*-KD (FDR < 0.05) in differentiated (**b**) gluteal and (**c**) abdominal DFAT cells. Genes with $\log_2$ fold-change of > 1 or < -1 are shown in turquoise. Top 20 genes with the most significant adjusted *p*-value are labelled. (**d**) Gene-set enrichment analysis results of DEGs in abdominal DFAT dox-induced *RSPO3*-KD cells showing the top 10 GO biological processes. Source data are provided as a Source Data file.

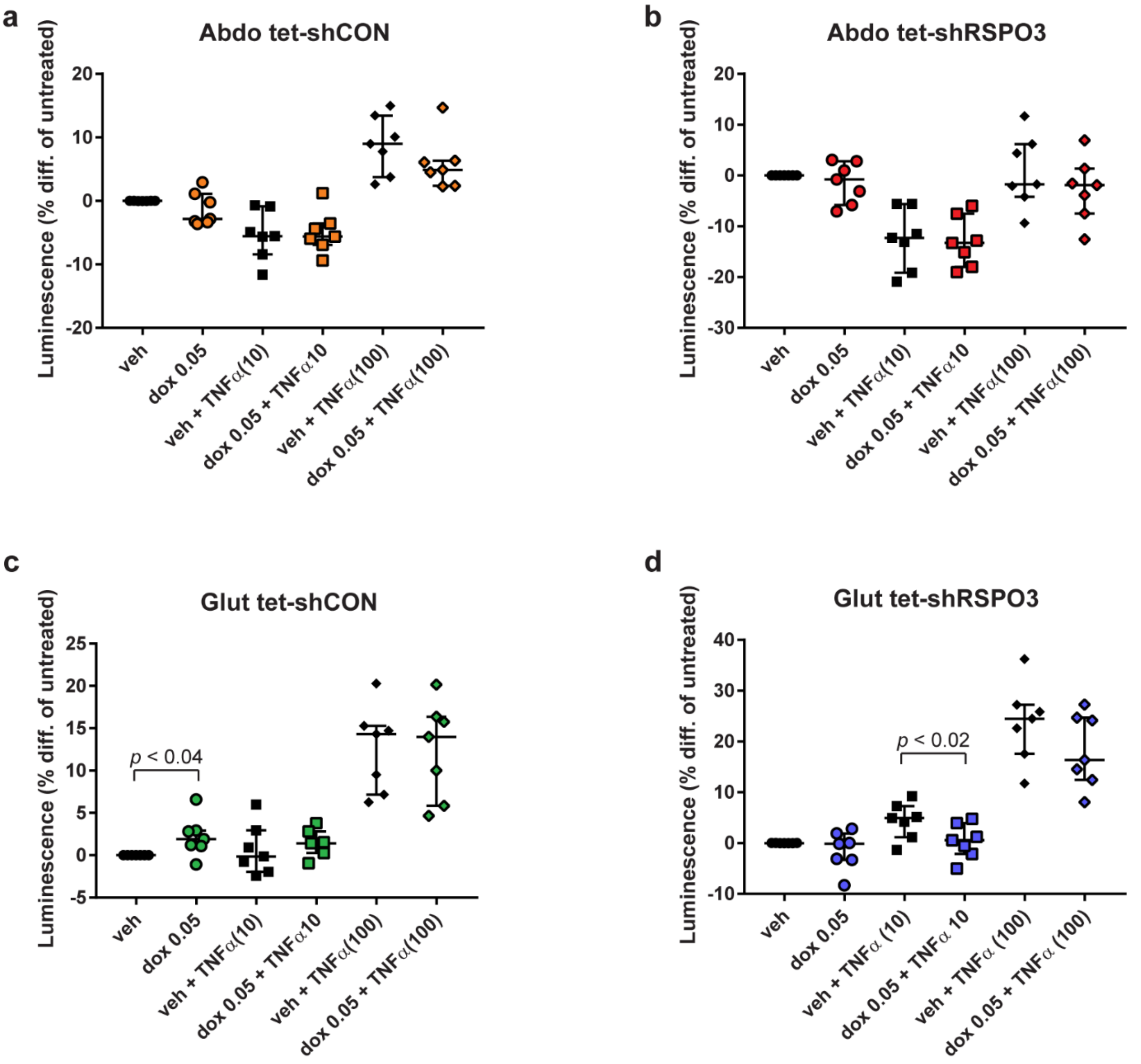

Supplementary Fig. 8. Effects of doxycycline-induced *RSPO3*-KD in abdominal and gluteal DFAT adipocytes on apoptosis in the presence of indicated concentrations of rhTNFα (ng ml^{-1}). Apoptosis was assayed using Caspase Glo 3/7 reagent. Results are shown as a % difference of luminescence of untreated cells (n = 7 independent experiments). Statistical significance was assessed by a two-tailed Wilcoxon signed-rank test comparing doxycycline (dox, 0.05 μg ml^{-1}) vs. non-dox (veh) treated cells in the presence of the same concentration of rhTNFα. Error bars are median values with interquartile ranges. Solid symbols, vehicle-treated; open symbols, doxycycline-treated; circles, no rhTNFα; squares, 10

ng ml^{-1} rhTNFα; diamonds, 100 ng ml^{-1} rhTNFα. Source data are provided as a Source Data file.

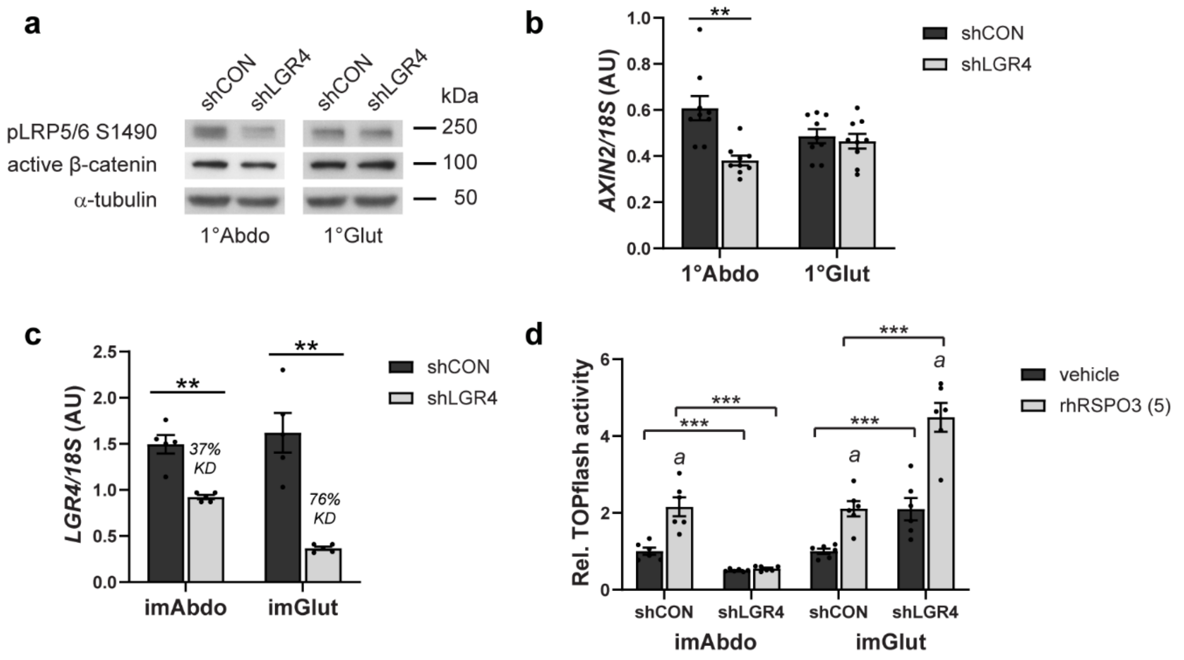

Supplementary Fig. 9. Effects of *LGR4*-KD in SC abdominal and gluteal APs on WNT signalling. *LGR4*-KD leads to reduced canonical WNT signalling in abdominal (Abdo), but not in gluteal (Glut), APs. (**a**) Western blots of pLRP5/6-S1490 and active β-catenin. (**b**) qRT-PCR analysis of *AXIN2* (n = 9 experiments). (**c-d**) Differential effects of *LGR4*-KD on TOPflash activity in imAbdo and imGlut cells. (**c**) *LGR4*-KD was confirmed by qRT-PCR in imAbdo and imGlut stably expressing 7TFC (n = 5 experiments). Effects of *LGR4*-KD in imAbdo and imGlut APs on TOPflash activity following treatment with vehicle or 5ng ml^{-1} rhRSPO3 (n = 6 replicates, representative of 6 experiments). Histogram data are means ± s.e.m.. $^{**}p < 0.01$, $^{***}p < 0.001$, shCON vs. shLGR4. $^{a}p < 0.01$, vehicle vs. rhRSPO3-treated. Statistical significance was assessed by two-tailed paired (**b**, **c**) and unpaired (**d**) Student's t-tests. Source data are provided as a Source Data file.

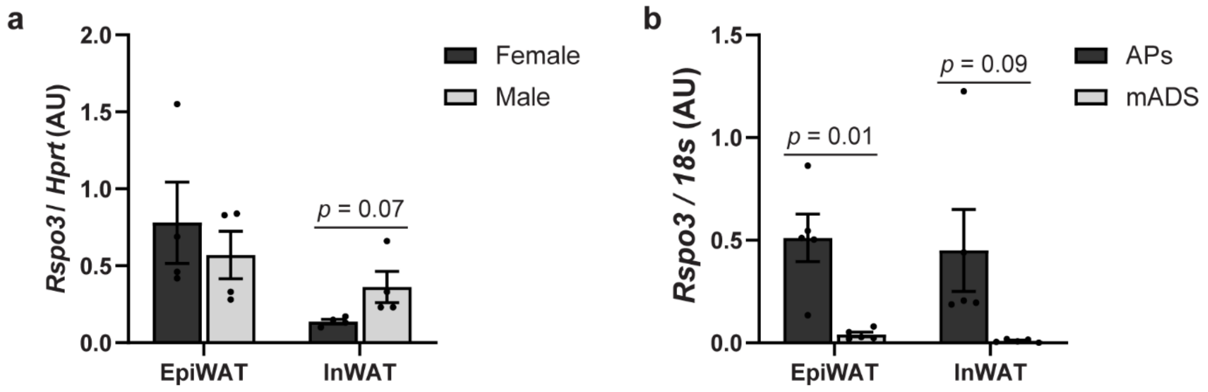

Supplementary Fig. 10. *Rspo3* expression in mouse whole AT and AT fractions. *Rspo3* expression was assess by qRT-PCR in (**a**) white adipose tissue (WAT) from epidydimal (Epi) and inguinal (In) fat from female and male mice (n = 4 each), and in (**b**) adipose tissue fractions [cultured APs and isolated mature adipocytes (mADS)] (n = 5 pairs), from epidydimal (Epi) and inguinal (In) fat. Histogram data are means ± s.e.m.. qRT-PCR data were normalised to *Hprt* (**a**) and *18s* (**b**). Statistical significance was assessed by two-tailed unpaired (**a**) and paired (**b**) Student's t-tests. Source data are provided as a Source Data file.

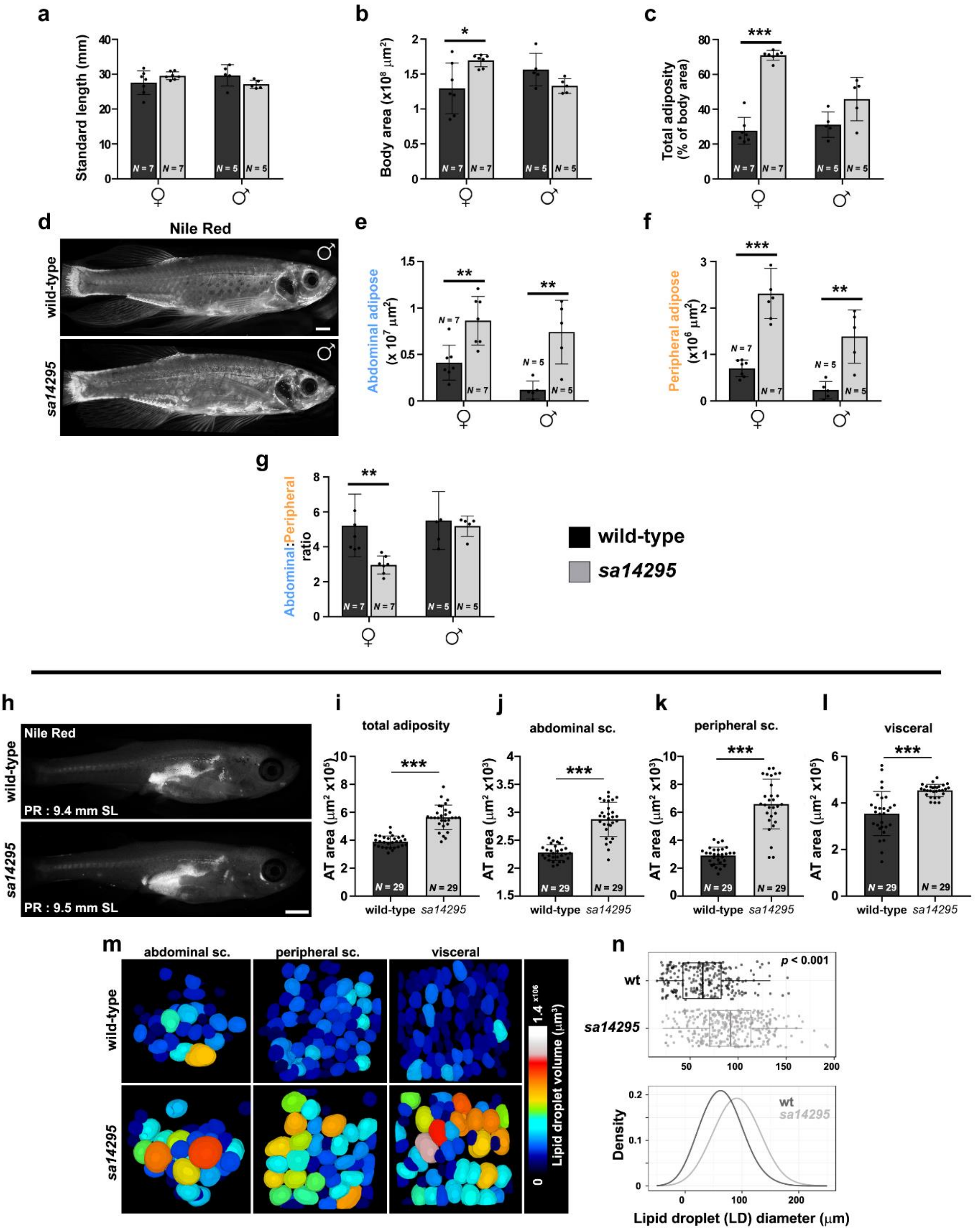

Supplementary Fig. 11. Effects of an *rspo3* nonsense mutation on adiposity in zebrafish. (**a-g**) Rspo3 has a sexually dimorphic effect on adiposity in adult zebrafish. (**a**) Standard length was not different between wild-type (wt) and *sa14295* homozygous (*rspo3*$^{m/m}$) males and females. (**b**) Body area was slightly increased in *rspo3*$^{m/m}$ females relative to wild-types, but

was unchanged between genotypes in males. (**c**) Assessment of generalized adiposity (AT area as a % of body area) revealed significant effect of genotype in females, but no change in males. (**d**) Nile Red images of wt and *rspo3*$^{m/m}$ males. Scale bar = 1 mm. (**e**) Abdominal adipose tissue was increased in both female and male mutants relative to wt siblings. (**f**) Peripheral adipose tissue was increased in both female and male mutants relative to wt siblings. (**g**) The ratio of abdominal to peripheral adipose tissues was decreased in *rspo3*$^{m/m}$ females, but not in *rspo3*$^{m/m}$ males relative to sex-matched wt siblings. Female wt siblings, $n = 7$; *rspo3*$^{m/m}$ females, $n = 7$; male wt siblings, $n = 5$; *rspo3*$^{m/m}$ males, $n = 5$. (**h-n**) Juvenile *rspo3*$^{m/m}$ zebrafish prior to overt sexual differentiation, have increased adiposity. (**h**) Nile Red staining of postembryonic 'PR' stage wt or *rspo3*$^{m/m}$ zebrafish. Scale bar = 1 mm. (**i-l**) Area quantification of total adiposity (**i**), abdominal SC adipose tissue (ASAT) (**j**), peripheral SC adipose tissue (LSAT) (**k**), and visceral adipose tissue (PVAT) (**l**). (**m**) Confocal imaging of lipid droplet size within 3 representative juvenile zebrafish ATs. Lipid droplets are coloured according to volume. (**n**) Peripheral SC lipid droplet diameter distributions. Box plots are shown in top panel, and a probability density function is shown in the lower panel. Box and whisker plot: center line, median; box limits, upper and lower quartiles; and whiskers, 1.5x of the interquartile range. Histogram data are means ± s.d.. *$p < 0.05$, **$p < 0.01$, ***$p < 0.001$. Two-tailed unpaired Student's t-tests were performed to assess differences between genotypes. Source data are provided as a Source Data file.

Supplementary Table 1. Associations between rs72959041 and rs1936807 and cardiometabolic traits

Trait	Dataset	PUBMED ID	rs72959041<br>EA = A			rs1936807<br>EA = G		
			β	*p*-value	*n*	*β*	*p*-value	*n*
Fasting Insulin	MAGIC [1]	22581228	NA	NA	NA	0.013	6×10^{-5}	51750
Fasting Insulin adj BMI	MAGIC [1]	22581228	NA	NA	NA	0.011	1×10^{-4}	51750
HOMA IR	MAGIC [2]	20081858	NA	NA	NA	0.012	0.003	37037
HDL Cholesterol	GLGC [3]	24097068	-0.038	0.0006	87274	-0.020	5×10^{-10}	184060
LDL Cholesterol	GLGC [3]	24097068	0.032	0.005	77651	0.003	0.6	170036
Triglycerides	GLGC [3]	24097068	0.044	2×10^{-7}	81266	0.015	1×10^{-5}	174722
T2D	DIAMANTE (European) [4]	30297969	0.044	0.002	231420	-0.017	0.008	231420
T2D adj BMI	DIAMANTE (European) [4]	30297969	0.064	0.0002	157384	-0.020	0.007	157384

Abbreviations: EA, effect allele; NA, data not available; BMI, body mass index; HOMA IR, Homeostatic Model Assessment for Insulin Resistance; HDL, high density lipoprotein; LDL, low density lipoprotein; SNV, single nucleotide variant; T2D, type 2 diabetes; T2D adj BMI, type 2 diabetes adjusted for BMI; WHRadjBMI, waist-hip ratio adjusted for BMI.

Supplementary Table 2. Anthropometric characteristics of subjects from whom paired abdominal and gluteal AT biopsies were obtained for gene expression studies

	**Female**	**Male**
n	105	102
Age (years)[a]	44.2 ± 0.5 (33, 53)	44.6 ± 0.4 (34, 53)
BMI (kg m^{-2})[a]	26.3 ± 0.5 (18.8, 43.3)	28.6 ± 0.5 (20.6, 46.2)

[a] mean ± s.e.m. (min, max). Abbreviations: AT, adipose tissue; BMI, body mass index.

Supplementary Table 3. Anthropometric characteristics of subjects heterozygous at rs72959041 from whom gDNA and AT cDNA were used for allelic-expression imbalance PCR studies

	**Female**	**Male**
n	7	7
Age (years)[a]	46 (36, 51)	44 (34, 53)
BMI (kg m^{-2})[a]	25 (21, 27)	28 (24, 40)

[a] median (min, max). Abbreviations: AT, adipose tissue; gDNA, genomic DNA; BMI, body mass index.

Supplementary Table 4. RSPO3 cis-eQTLs are limited to adipose tissue

Tissue	Source	Platform	*n*	rs72959041	rs9491696
				Cis eQTL p-value	Cis eQTL p-value
Leg AT	GTex [5]	RNA seq	385	4.0E-07	8.07E-02
Visceral AT	GTex [5]	RNA seq	313	9.2E-01	9.05E-01
Subcutaneous Abdominal AT	TwinsUK [6]	RNA seq	766	4.05E-15	4.49E-11
Subcutaneous Abdominal AT	METSIM [7]	Microarray	770	3.0E-28	9.7E-10
Subcutaneous Abdominal AT	deCODE [8, 9]	Microarray	673	3.2E-17	1.1E-7
Skin	TwinsUK [10]	Microarray	667	NA	0.01
Lymphoblastoid cell lines	TwinsUK [10]	Microarray	777	NA	0.28

Abbreviations: AT, adipose tissue; NA, data not available; SNV, single nucleotide polymorphism; WHRadjBMI, waist-hip ratio adjusted for BMI.

.

Supplementary Table 5. Anthropometric characteristics of subjects heterozygous at rs72959041 from whom gDNA, and mature adipocyte and AP cDNA were used for allelic-expression imbalance PCR studies

	**Female**	**Male**
n	10	3
Age (years)[a]	50 (43, 67)	41 (34, 53)
BMI (kg m^{-2})[a]	25 (21, 33)	24 (21, 33)

[a] median (min, max). Abbreviations: AP, adipose progenitors; gDNA, genomic DNA; BMI, body mass index.

Supplementary Table 6. Anthropometric characteristics of subjects heterozygous at rs9491696 from whom gDNA and AT cDNA were used for allelic-expression imbalance PCR studies

	**Female**	**Male**
n	15	17
Age (years)[a]	45 (33, 50)	45 (34, 52)
BMI (kg m^{-2})[a]	24 (19, 36)	28 (23, 41)

[a] median (min, max). Abbreviations: AT, adipose tissue; gDNA, genomic DNA; BMI, body mass index.

Supplementary Table 7. Anthropometric characteristics of subjects heterozygous at rs9491696 from whom gDNA, and mature adipocyte and AP cDNA were used for allelic-expression imbalance PCR studies

	**Female**	**Male**
n	19	22
Age (years)[a]	43 (31, 67)	40 (34, 67)
BMI (kg m^{-2})[a]	26 (22, 40)	25 (22, 33)

[a] median (min, max). Abbreviations: AP, adipose progenitors; gDNA, genomic DNA; BMI, body mass index.

Supplementary Table 8. Anthropometric characteristics of subjects from whom histology data were obtained for adipocyte cell sizing

	**Female**		
**rs72959041 genotype**	**GG**	**GA**	**p-value**
n	15	15	
Age (years)[a]	50.7 (43.6, 65.5)	49.9 (44.6, 67)	0.9
BMI (kg m^{-2})[a]	25.6 (22.0, 33.9)	27.0 (21.1, 32.6)	0.8

[a] median (min, max). rs72959041-GG subjects were matched with rs72959041-GA subjects on age (within 3 years), sex (absolute) and BMI (within 2 kg m^{-2}). Statistical difference between groups was assessed using a two-tailed Wilcoxon signed-rank test. Abbreviation: BMI, body mass index.

Supplementary Table 9. Anthropometric characteristics of female subjects from whom DXA scans and paired abdominal and gluteal AT biopsies were obtained for gene expression studies

	**All**	**With DXA**
n	61	43
Age (years)[a]	46.9 ± 1.2 (30.8, 67.4)	50.7 ± 1.2 (37.5, 67.4)
BMI (kg m^{-2})[a]	27.7 ± 0.5 (22.1, 40)	27.4 ± 0.6 (22.1, 35.3)

[a] mean ± s.e.m. (min, max). Abbreviations: AT, adipose tissue; DXA, dual-energy X-ray absorptiometry; BMI, body mass index.

Supplementary Table 10. TaqMan SNP genotyping and gene expression assays, and primers used for qRT-PCR

SNP/Gene	Assay ID/primer sequence
rs577721086	C_397393717_10
rs1892172	C____270711_20
18S	Hs99999901_s1
RSPO3	Hs00262176_m1
ADIPOQ	Hs00605917_m1
AXIN2	Hs00610344_m1
CEBPA	Hs00269972_s1
FABP4	Hs00609791_m1
HOTAIR	Hs03296680_s1
HOXA5	Hs00430330_m1
HOXA6	Hs00430615_m1
LGR4	Hs00173908_m1
PGK1	Hs99999906_m1
PLIN1	Hs00160173_m1
PNPLA2	Hs00386101_m1
PPARG2	Hs01115510_m1
PPIA	Hs99999904_m1
SHOX2	Hs00243203_m1
Rspo3 (mouse)	Mm01188251_m1
18s (mouse)	Mm03928990_g1
Hprt (mouse)	Mm03024075_m1
rspo3 (zebrafish)	5’-AGATGCTGCTCCTCATTGCT<br>5’-CTGGCCCCTGTTACACAGTT
18S (zebrafish)	5’-CACTTGTCCCTCTAAGAAGTTGCA<br>5’-GGTTGATTCCGATAACGAACGA
pparg (zebrafish)	5’-TGCCGCATACACAAGAAGAG<br>5’-ATGTGGTTCACGTCACTGGA

Supplementary Methods

RNA-seq library preparation and analysis

DFAT tet-shCON and tet-shRSPO3 cells were differentiated by standard differentiation protocol. On day 13 of differentiation, cells were treated doxycycline (final concentration of 0.05 μg ml^{-1}), or vehicle, in hormone-free basal media, for two days. On day 15, cells were harvested for RNA. RNA-seq was performed on samples from three independent experiments. Total RNA purification and on-column DNaseI-treatment were performed using the RNeasy Mini kit (Qiagen). RNA concentration was assessed using the NanoDrop ND-1000 (Labtech) and RiboGreen (Invitrogen) on the FLUOstar OPTIMA plate reader (BMG Labtech), and RNA quality using the Agilent 2100 Bioanalyzer (Agilent) and the 2200 or 4200 TapeStation (Agilent, RNA ScreenTape). RNA integrity number (RIN) estimates for all samples were between 7-10. Library preparation, cDNA sequencing and analysis were performed at the Oxford Genomics Centre (Wellcome Trust Centre for Human Genetics, Oxford, UK). Polyadenylated transcript enrichment and strand specific library preparation was completed using NEBNext Ultra II mRNA kit (NEB) following manufacturer's instructions. Libraries were amplified on a Tetrad (Bio-Rad) using in-house unique dual indexing primers (based on DOI: 10.1186/1472-6750-13-104). Individual libraries were normalised using Qubit, and the size profile was analysed on the 2200 or 4200 TapeStation. Individual libraries were normalised and pooled together accordingly. The pooled library was diluted to ~10 nM for storage. The 10 nM library was denatured and further diluted prior to loading on the sequencer. Paired end sequencing was performed using a HiSeq4000 75bp platform (Illumina, HiSeq 3000/4000 PE Cluster Kit and 150 cycle SBS Kit), generating a raw read count of 20-25 million reads per sample. The sequencing quality was assessed with FASTQC (http://www.bioinformatics.babraham.ac.uk/projects/fastqc/). Reads were aligned to the human reference genome (GRCh37) using HISAT2[11] and duplicate reads removed using the

Picard 'MarkDuplicates' tool (http://broadinstitute.github.io/picard). Reads mapping uniquely to Ensembl-annotated genes were summarised using featureCounts[12]. The raw gene count matrix was imported into the R/BioConductor environment (http://www.R-project.org)[13] for further processing and analysis with the edgeR package[14]. Genes with very low expression were excluded based on the following heuristic: to be retained a gene needed to be expressed at the equivalent of 10 reads or more (after normalising for library size) in at least as many samples as the smallest experimental group considered (in this case, usually 3). Multiple testing correction was performed by using edgeR's default Benjamini-Hochberg method for controlling the false discovery rate (FDR). Gene-set enrichment analysis of differentially expressed genes (FDR < 0.05) was performed in Metascape[15].

Mouse husbandry

Inbred laboratory mice C57BL/6N were housed at the Mary Lyon Centre (Harwell, UK) in accordance with UK Home Office legislation and local ethical guidelines issued by the Medical Research Council, UK. Mice were maintained under controlled light (12-hour light and dark cycle), temperature (21 ± 2°C) and humidity (55 ± 10%). Mice had free access to water (10-13 ppm chlorine) and were fed ad libitum on a commercial diet (Rat and Mouse No. 3 breeding diet [RM3], Special Diets Services, UK).

Supplementary References

1. Manning, A. K., et al. A genome-wide approach accounting for body mass index identifies genetic variants influencing fasting glycemic traits and insulin resistance. *Nat Genet* **44**, 659-669 (2012).

2. Dupuis, J., et al. New genetic loci implicated in fasting glucose homeostasis and their impact on type 2 diabetes risk. *Nat Genet* **42**, 105-116 (2010).

3. Willer, C. J., et al. Discovery and refinement of loci associated with lipid levels. *Nat Genet* **45**, 1274-1283 (2013).

4. Mahajan, A., et al. Fine-mapping type 2 diabetes loci to single-variant resolution using high-density imputation and islet-specific epigenome maps. *Nat Genet* **50**, 1505-1513 (2018).

5. Consortium, G. T., et al. Genetic effects on gene expression across human tissues. *Nature* **550**, 204-213 (2017).

6. Buil, A., et al. Gene-gene and gene-environment interactions detected by transcriptome sequence analysis in twins. *Nat Genet* **47**, 88-91 (2015).

7. Civelek, M., et al. Genetic Regulation of Adipose Gene Expression and Cardio-Metabolic Traits. *Am J Hum Genet* **100**, 428-443 (2017).

8. Styrkarsdottir, U., et al. Sequence variants in the PTCH1 gene associate with spine bone mineral density and osteoporotic fractures. *Nat Commun* **7**, 10129 (2016).

9. Heid, I. M., et al. Meta-analysis identifies 13 new loci associated with waist-hip ratio and reveals sexual dimorphism in the genetic basis of fat distribution. *Nat Genet* **42**, 949-960 (2010).

10. Grundberg, E., et al. Mapping cis- and trans-regulatory effects across multiple tissues in twins. *Nat Genet* **44**, 1084-1089 (2012).

11. Kim, D., Langmead, B. & Salzberg, S. L. HISAT: a fast spliced aligner with low memory requirements. *Nat Methods* **12**, 357-360 (2015).

12. Liao, Y., Smyth, G. K. & Shi, W. featureCounts: an efficient general purpose program for assigning sequence reads to genomic features. *Bioinformatics* **30**, 923-930 (2014).

13. Gentleman, R. C., et al. Bioconductor: open software development for computational biology and bioinformatics. *Genome Biol* **5**, R80 (2004).

14. Robinson, M. D., McCarthy, D. J. & Smyth, G. K. edgeR: a Bioconductor package for differential expression analysis of digital gene expression data. *Bioinformatics* **26**, 139-140 (2010).

15. Zhou, Y., et al. Metascape provides a biologist-oriented resource for the analysis of systems-level datasets. *Nat Commun* **10**, 1523 (2019).
