# Supplementary material for: RSPO3 impacts body fat distribution and regulates adipose cell biology in vitro
Source: Nat Commun. 2020 Jun 3;11:2797. doi: 10.1038/s41467-020-16592-z (PMC7271210; doi:10.1038/s41467-020-16592-z)
Supplement: Supplementary file 3 — Description of Additional Supplementary Information [file 41467_2020_16592_MOESM3_ESM.pdf]

Description of Additional Supplementary Files

File Name: Supplementary Data 1
Description: GWAS 99% credible sets. RSPO3 WHRadjBMI signals co-localise with subcutaneous adipose tissue cis-eQTLs.
