# Supplementary material for: RSPO3 impacts body fat distribution and regulates adipose cell biology in vitro
Source: Nat Commun. 2020 Jun 3;11:2797. doi: 10.1038/s41467-020-16592-z (PMC7271210; doi:10.1038/s41467-020-16592-z)
Supplement: Supplementary file 5 — Reporting Summary [file 41467_2020_16592_MOESM5_ESM.pdf]

nature research

Corresponding author(s): Constantinos Christodoulides & Fredrik Karpe

Last updated by author(s): 2020-4-24

Reporting Summary

Nature Research wishes to improve the reproducibility of the work that we publish. This form provides structure for consistency and transparency in reporting. For further information on Nature Research policies, see Authors & Referees and the Editorial Policy Checklist .

Statistics

For all statistical analyses, confirm that the following items are present in the figure legend, table legend, main text, or Methods section.

n/a	Confirmed	
☐	☒	The exact sample size (*n*) for each experimental group/condition, given as a discrete number and unit of measurement
☐	☒	A statement on whether measurements were taken from distinct samples or whether the same sample was measured repeatedly
☐	☒	The statistical test(s) used AND whether they are one- or two-sided<br>*Only common tests should be described solely by name; describe more complex techniques in the Methods section.*
☐	☒	A description of all covariates tested
☐	☒	A description of any assumptions or corrections, such as tests of normality and adjustment for multiple comparisons
☐	☒	A full description of the statistical parameters including central tendency (e.g. means) or other basic estimates (e.g. regression coefficient) AND variation (e.g. standard deviation) or associated estimates of uncertainty (e.g. confidence intervals)
☐	☒	For null hypothesis testing, the test statistic (e.g. *F*, *t*, *r*) with confidence intervals, effect sizes, degrees of freedom and *P* value noted<br>*Give P values as exact values whenever suitable.*
☐	☒	For Bayesian analysis, information on the choice of priors and Markov chain Monte Carlo settings
☒	☐	For hierarchical and complex designs, identification of the appropriate level for tests and full reporting of outcomes
☐	☒	Estimates of effect sizes (e.g. Cohen's *d*, Pearson's *r*), indicating how they were calculated

Our web collection on statistics for biologists contains articles on many of the points above.

Software and code

Policy information about availability of computer code

Data collection	enCORE v14.1, IMPUTE2, Adobe Photoshop 5.0.1 , Image Processing Tool Kit (Reindeer Games, Gainesville, FL), ImageJ (v1.50a), Veritas Microplate Luminometer, CytoFluor Multi-well Plate Reader series 4000 (PerSeptive Biosystems), an EnSpire 2300 Multilabel Reader (Perkin Elmer), ILAB 650 clinical analyser (Instrumentation Laboratory UK), Beckman LS6500 Multipurpose Scintillation Counter (Beckman), Cellometer Auto T4 (Nexcelom Bioscience)
Data analysis	SPSS 22, Sigmaplot 14.0, Graphpad 7.04, PLINK v.1.07, FASTQC, HISAT2, Picard 'MarkDuplicates' tool, featureCounts, R version 3.6.1, edgeR, Metascape, R package 'coloc', GCTA, Minimac 3 on the Michigan Imputation Server , lme4 package in R, MatrixeQTL package in R version 3.5.0

For manuscripts utilizing custom algorithms or software that are central to the research but not yet described in published literature, software must be made available to editors/reviewers. We strongly encourage code deposition in a community repository (e.g. GitHub). See the Nature Research guidelines for submitting code & software for further information.

Data

Policy information about availability of data

All manuscripts must include a data availability statement. This statement should provide the following information, where applicable:

- Accession codes, unique identifiers, or web links for publicly available datasets
- A list of figures that have associated raw data
- A description of any restrictions on data availability

Full TwinsUK RNA-seq expression data from subcutaneous adipose tissue are available by direct application to TwinsUK. Full GWAS summary statistics from the UK Biobank meta-analysis for WHRadjBMI can be found on https://doi.org/10.5281/zenodo.1251813. The source data underlying Figs. 1b, d, e, g-m, 2, 3a, b, d-i, k-m, 4b-k, 5, 6b, d-g, i, Supplementary Figs. 1, 2, 3a-c, 4a, b, d-f, 5, 6, 7b-d, 8, 9, 10, 11a-c, e-g, i-l, n, and Table 2 are provided as a Source Data file. RNA-seq data that support the findings of this study have been deposited in GEO with the accession code GSE149294.
All other relevant data are available from the corresponding authors upon reasonable request.

Field-specific reporting

Please select the one below that is the best fit for your research. If you are not sure, read the appropriate sections before making your selection.

- [x] Life sciences
- [ ] Behavioural & social sciences
- [ ] Ecological, evolutionary & environmental sciences

For a reference copy of the document with all sections, see nature.com/documents/nr-reporting-summary-flat.pdf

Life sciences study design

All studies must disclose on these points even when the disclosure is negative.

Sample size	No formal sample size calculation was performed. Sample size for human and in vitro functional studies were based on publications of similar studies by Small et al. Nat. Genet. 50, 572–580 (2018), and Loh et al. Cell Metab. 21, 262-273 (2015), and our own research experience. It was also dependent on EAF and ~ 30% OBB volunteer recruitment success (for fat biopsies), availability of genotype and DXA data from the Oxford Biobank. Sample size for zebrafish studies was based on Minchin et al. Proc Natl Acad Sci U S A 112, 4363-4368 (2015).
Data exclusions	No data was excluded apart from extreme outliers for technical replicates for qRT-PCR (>= 1 cycle difference).
Replication	All in vitro studies were replicated in at least 3 independent experiments, unless otherwise stated. In the case where an experiment was only replicated twice, the results were conclusive. Replicate experiments were successful.
Randomization	There was no randomization. Genetic modification and/or treatment of cells were known prior to experiments. Equal number of replicates for each cell type and treatment condition was used.
Blinding	Data collected from human participants were blinded to genotype. Investigators were blinded to genotypes for cell sizing experiment. There was no blinding for in vitro cellular or zebrafish studies. Equal numbers of each group were tested.

Reporting for specific materials, systems and methods

We require information from authors about some types of materials, experimental systems and methods used in many studies. Here, indicate whether each material, system or method listed is relevant to your study. If you are not sure if a list item applies to your research, read the appropriate section before selecting a response.

Materials & experimental systems

n/a	Involved in the study	
[ ]	[x]	Antibodies
[ ]	[x]	Eukaryotic cell lines
[x]	[ ]	Palaeontology
[ ]	[x]	Animals and other organisms
[ ]	[x]	Human research participants
[x]	[ ]	Clinical data

Methods

n/a	Involved in the study	
[x]	[ ]	ChIP-seq
[x]	[ ]	Flow cytometry
[x]	[ ]	MRI-based neuroimaging

Antibodies

Antibodies used	Cell Signalling Technology: phospho-LRP5/6-S1490 rabbit pAb (#2568), phospho-JNK (Thr183/Tyr185) rabbit pAb (#9251); Merck Millipore: active-beta-catenin mouse mAb (05-665)]; Santa Cruz Biotechnology: total JNK mouse mAb (sc-7345), phospho-CaMKII-alpha (Thr286) rabbit pAb (sc12886-R), total CaMKII rabbit pAb (sc-9035); Abcam: alpha-tubulin rabbit pAb (ab15246); DAKO: HRP-conjugated goat anti-mouse IgG pAb (P0447); HRP-conjugated goat anti-rabbit IgG pAb (P0448).
Validation	The phospho-LRP6-S1490 rabbit pAb (#2568) has been used in over 100 publications, please see https://www.cellsignal.co.uk/products/primary-antibodies/phospho-lrp6-ser1490-antibody/2568. The phospho-JNK (Thr183/Tyr185) rabbit pAb (#9251) has been used in over 1000 peer-reviewed papers, please see www.cellsignal.co.uk/products/primary-antibodies/phospho-sapk-jnk-thr183-tyr185-antibody/9251. The active-beta-catenin mouse mAb (05-665) has been used in numerous publications, please see https://www.merckmillipore.com/GB/en/product/Anti-Active-Catenin-Anti-ABC-Antibody-clone-8E7,MM_NF-05-665. The total JNK mouse mAb (sc-7345) has been cited in 417 publications, see https://www.scbt.com/p/jnk-antibody-d-2. The phospho-CaMKII-alpha (Thr286) rabbit pAb (sc12886-R) has been cited in 18 publications, see https://www.scbt.com/p/p-camkiialpha-antibody-thr-286. The CaMKII rabbit pAb (sc-9035) has been cited in 29 publications, please see https://www.scbt.com/p/camkii-antibody-m-176. The alpha-tubulin rabbit pAb (ab15246) is an antibody that has been extensively used including the 166 publications that are found on https://www.abcam.com/alpha-tubulin-antibody-microtubule-marker-ab15246.html.

Eukaryotic cell lines

Policy information about cell lines

Cell line source(s)	HEK293 (ATCC, CRL-1573). All other cell lines used in the manuscript were generated in-house by immortalisation of human abdominal and gluteal adipose progenitors derived from fat biopsies with human telomerase reverse transcriptase and HPV-E7 oncoprotein.
Authentication	We have used gene expression signatures (by Taqman PCR) to authenticate the depot origin of the adipose cell lines. Additionally we have undertaken transcriptional profiling of DFAT cells using RNA-seq.
Mycoplasma contamination	Confirm. All cell-lines have been tested routinely and confirmed to be free of mycoplasma contamination.
Commonly misidentified lines (See ICLAC register)	none

Animals and other organisms

Policy information about studies involving animals; ARRIVE guidelines recommended for reporting animal research

Laboratory animals	Zebrafish: Animals were obtained from the Wellcome Trust Sanger Institute Zebrafish Mutation Project as F3 embryos after out-crossing to Hubrecht long fin wild type zebrafish in the previous generation. F3 adults were subsequently out-crossed to the Ekkwill wild type strain and F4 carriers inter-crossed for experiments. Experiments were conducted on 5 month-old male and female zebrafish, and postembryonic zebrafish aged 21 or 26 days post-fertilisation. Mice: C57BL/6N. Tissue samples were collected from 8-10 week-old male and female mice for whole adipose tissue, and from male mice for fractionated adipose tissue.
Wild animals	No wild animals were used in the study.
Field-collected samples	No field-collected samples were used in the study.
Ethics oversight	All zebrafish experiments conformed to the U.S. Public Health Service Policy on Humane Care and Use of Laboratory Animals, using protocols approved by the Institutional Animal Care and Use Committee of Duke University. Mice were housed at the Mary Lyon Centre (Harwell, UK) in accordance with UK Home Office legislation and local ethical guidelines issued by the Medical Research Council, UK.

Note that full information on the approval of the study protocol must also be provided in the manuscript.

Human research participants

Policy information about studies involving human research participants

Population characteristics	The Oxford BioBank (OBB) comprises a randomised, age-stratified sample obtained from Oxfordshire and the Thames Valley in the U.K.. The Thames Valley Primary Care Agency has enabled random recruitment by providing lists of Oxfordshire residents registered with a local general practitioner and aged 30–50 years. Individuals with a previous diagnosis of myocardial infarction or heart failure currently on treatment; untreated malignancy; other ongoing systemic diseases, and pregnant women were excluded from participation. The OBB recruitment began in 1999 and included 7640 individuals (4316 women and 3324 men) as of October 2016. OBB subjects were genotyped on the Illumina Human Exome BeadChip and Affymetrix UK Biobank Axiom arrays. Genotype imputation was performed using the Affymetrix UK Biobank Axiom array with Haplotype Reference Consortium (HRC), 1000Genome and UK10K reference panels using IMPUTE2 software. (Karpe et al. Int J Epidemiol. 47, 21-21g (2018)).<br>UK Biobank is a prospective study with over 500,000 participants (male and female) aged 40–69 years when recruited in 2006–2010 from 22 assessment centers throughout the UK, with medical history, genetic information and anthropometric data available (Sudlow et al. PLoS Med. 12, e1001779 (2015)).<br>The TwinsUK Adult twin registry comprise over 14,000 volunteer twins (monozygotic, dizygotic and triplets, 82% females, mean age 59 years) with genetic information, and RNA-seq expression data from subcutaneous adipose tissue for a total of 856 female twins. After QC, 766 adipose RNA-seq samples were available for analysis, of which 720 had available genotypes. The female donors of the TwinsUK adipose samples had a median age of 60 years (38–84 years) and median BMI of 25 kg/m2 (Grundberg et al., Nat Genet. 44, 1084-1089 (2012); Glastonbury et al. Am J Hum Genet. 104, 1013-1024 (2019)).
Recruitment	An invitation letter along with the study information and response sheet were sent to all participants. Individuals who expressed willingness to enroll in the OBB were contacted by telephone or email, in order to convey a brief overview of the study aims and objectives, by trained research nurses.
Ethics oversight	All studies were approved by the Oxfordshire Clinical Research Ethics Committee and all volunteers gave written, informed consent.

Note that full information on the approval of the study protocol must also be provided in the manuscript.
